# Lithotomy

**Published:** 1867-06

**Authors:** D. W. Hammond

**Affiliations:** Macon, Ga.


### ARTICLE III.

*Lithotomy.* By D. W. Hammond, M. D., Macon, Ga.

W. T. Nelson, aged forty-one, a merchant of this city, has had vesical trouble for three years.

For the last six months his sufferings have been unremittingly severe, finding no relief except when under large doses of opium or morphine. He was sounded sometime during last year, by Dr. Mettaeur, and a calculus was detected: shortly after which, he placed himself under my care. I gave him the usual palliatives in such cases, urging him all the while to submit to the operation, it being, "*ultimum et unicum remedium*," for his alleviation. On the 4th April, 1867, he came to my office, and informed me that he had come to the determination to have the calculus extracted, as he could not endure the pain any longer. I directed him to go home and take a dose of ol. ricini, and that I would operate next morning, at 10 o'clock. When the time arrived, the following physicians, viz., Boon, Mettaeur, Castlin, Holt, Smith, Mason, Blackshear, and Branham, kindly gave me their council and assistance. Having secured him in the usual position, my friend Dr. Castlin administered chloroform; when fully anæsthetised, my friend Dr. Mettauer, by request, introduced a No. 10 grooved director into the bladder. The existence of the calculus being unmistakable, I at once made the perinæal section, and with a ¾ inch gorget, as modified by Dr. Physic, the prostate gland and neck of the bladder were divided. The calculus could be distinctly felt lying in the bas-fond of the viscus. Finding it to be small, its removal was attempted by the introduction of the scoop: this failing to extract it, several different shaped forceps were used, and finally it was grasped, and in bringing it through the neck of the bladder, it broke, and fell in several pieces. The operation now became tedious and somewhat embarrassing, being compelled to make repeated attempts to remove the remaining fragments. But by perseverance with divers instruments, and injecting water into the bladder, it was finally completely rid of the detritus. As advised by Liston, a No. 10 bougie was inserted, and pledgets of lint packed around it, as there was some hæmorrhage still oozing from the wound. The external pubic artery was large, and when cut, the blood sprang out with considerable force, which, however, ceased to bleed before the completion of the operation. The patient was now removed from the table and placed in bed, and ʒi. tr. opii administered; pulse 100, and rather feeble; gave him a drink of whisky and water. At 7 P. M., restless, and had a constant desire to void urine, which was passing partly through the bougie, and partly per urethram. Supposing that this uncontrollable desire to discharge urine was the result of the presence of the bougie and the packing around it, I withdrew them, as the hæmorrhage had entirely subsided. Ordered an anodyne draught, and left him for the night.

6th—Saw him at 9 A. M.; found him comparatively comfortable; pulse 105; urine passing almost entirely through the penis; directed flax seed tea as a constant drink. 6 P. M., still in great pain from constant desire to empty the bladder; urine bloody, and passing through the urethra. The wound was agglutinated and glazed over, and had every appearance of uniting by the first intention. Fearing this, I made a slight effort to pull it open; but the adhesion was so firm, and gave him so much pain, I desisted. I will here mention, that many years ago I operated upon a small lad for stone, and the wound healed by the first intention, and twelve or fourteen days afterwards a perinæal abscess formed, which broke in a few days, the result of which, was a troublesome fistulous opening, requiring over two months to heal.

7th.—Had a comfortable night, and feeling tranquil this morning; no fever; pulse 90; and to my gratification the urine had resumed its route through the perenæum. Ordered a dose of ol. ricini, which operated through the day. 9 P. M., advised one gr. sulpt. morphine by enema.

8th.—Rested badly through the night, otherwise doing well; appetite returning; urine passing both ways.

9th.—Spent a comfortable day; pulse 85; more cheerful; urine discharged mostly through the urethra; wound closing rapidly; directed an enema of morphine to be administered at night.

10th.—Doing well; pulse 78; but little pain in voiding urine.

From the 11th to the 14th, continued to improve.

The wound closed, and the urine discharged without any obstruction or pain *per vias naturales.* By the 18th, (13 days after the operation) he was in his store-room attending to business, and on the 20th, went with some friends to the lakes below the city fishing. Calculous about the size of a large almond; weight, 96 grs.

